# Temporary Mechanical Circulatory Support: Left, Right, and Biventricular **Devices**

**DOI:** 10.2174/1573403X19666230314115853

**Published:** 2023-07-17

**Authors:** Michael Dangl, Michael Albosta, Hoda Butros, Matthias Loebe

**Affiliations:** 1 Department of Internal Medicine, University of Miami Miller School of Medicine/Jackson Memorial Hospital, Miami, FL, USA;; 2 Department of Medicine, Cardiovascular Division, University of Miami Miller School of Medicine/Jackson Memorial Hospital, Miami, FL, USA;; 3 Department of Surgery, Division of Cardiothoracic Surgery, University of Miami Miller School of Medicine/Jackson Memorial Hospital, Miami, FL, USA

**Keywords:** Heart failure, mechanical circulatory support, cardiogenic shock, left ventricular assist device, intra-aortic balloon pump, ECMO, impella

## Abstract

Temporary mechanical circulatory support (MCS) encompasses a wide array of invasive devices, which provide short-term hemodynamic support for multiple clinical indications. Although initially developed for the management of cardiogenic shock, indications for MCS have expanded to include prophylactic insertion prior to high-risk percutaneous coronary intervention, treatment of acute circulatory failure following cardiac surgery, and bridging of end-stage heart failure patients to more definitive therapies, such as left ventricular assist devices and cardiac transplantation. A wide variety of devices are available to provide left ventricular, right ventricular, or biventricular support. The choice of a temporary MCS device requires consideration of the clinical scenario, patient characteristics, institution protocols, and provider familiarity and training. In this review, the most common forms of left, right, and biventricular temporary MCS are discussed, along with their indications, contraindications, complications, cannulations, hemodynamic effects, and available clinical data.

## 1 INTRODUCTION

Temporary mechanical circulatory support (MCS) encompasses a wide array of invasive devices, which provide short-term hemodynamic support for multiple clinical indications. Although initially developed for the management of cardiogenic shock (CS), indications for MCS have expanded to include prophylactic insertion prior to high-risk percutaneous coronary intervention (PCI), treatment of acute circulatory failure following cardiac surgery, and bridging of end-stage heart failure patients to more definitive therapies, such as left ventricular assist devices (LVAD) and cardiac transplantation (Table **1**).

Cardiogenic shock remains the most common indication for temporary MCS use and is manifested by clinical criteria of end-organ malperfusion. Systolic blood pressure <90mmHg for more than 30 minutes despite fluid resuscitation or use of inotropes, a cardiac index of <2.2 L/min/m2, or pulmonary capillary wedge pressure >15mmHg define cardiogenic shock. CS is recognized as a spectrum of diseases ranging from mild hypoperfusion to profound shock in patients at risk for developing CS to those refractory to multiple therapies. An Expert Consensus Statement from the Society for Cardiovascular Angiography and Interventions (SCAI) elaborated on the spectrum of disease by proposing a new CS staging scheme, from A to E, as detailed in Table **2**. Although multiple hemodynamic parameters can be utilized to monitor cardiac function, the strongest hemodynamic correlate of mortality in patients with cardiogenic shock is the cardiac power output (CPO), calculated as mean arterial pressure x cardiac output /451 [1]. A CPO cutoff of 0.53 Watts was found to best delineate the risk of in-hospital mortality, with a CPO ≤0.53 W associated with a 58% risk of in-hospital mortality and a CPO >0.53 W associated with a 29% risk of in-hospital mortality (c-statistic = 0.69) [1].

Acute myocardial infarction is the most common cause of CS, accounting for approximately 81% of cases [2]. Additional etiologies include decompensated non-ischemic heart failure, severe valvular stenosis /regurgitation, rupture of the papillary muscle or chordae tendinae, tachy- and brady-arrhythmias, myocarditis, ventricular septal defects and ventricular free wall aneurysms (Table **3**). Pathology can affect the left ventricle, right ventricle, or both ventricles simultaneously. As a result, cardiogenic shock can result from left, right, or bi-ventricle failure.

In the initial 1999 SHOCK trial, mortality from cardiogenic shock was >40% [3]. Despite advancements in the treatment of cardiogenic shock and the introduction of new mechanical support devices, limited progress has been made in reducing the mortality due to cardiogenic shock. In 2016, after the approval of the Impella temporary mechanical circulatory support device, the SHOCK trial investigators initiated the “Detroit Cardiogenic Shock Initiative” and developed a CS protocol that emphasized: 1) Early identification of CS and catheterization lab activation, 2) Early initiation of MCS (prior to PCI or inotropes), 3) Routine use of invasive hemodynamic monitoring, and 4) Limiting device-related complications [4]. Initial results from implementing the protocol revealed decreased hospital mortality to 28%, highlighting the benefit of early temporary mechanical support.

In 2018, the United Network of Organ Sharing (UNOS) updated the allocation policy for heart transplantation in the United States in an effort to reduce the mortality of those on the waiting list [5]. In the new policy, a higher priority is given to patients on extra-corporeal membrane oxygenation and other temporary mechanical support devices and lower priority is given to patients on durable left ventricular assist devices. The reasoning behind this new allocation policy is the high mortality associated with ECMO and other short-term mechanical circulatory devices and advances and improved clinical outcomes with LVAD, leading to a less pressing need for heart transplantation. However, there is concern that this policy change will provide an incentive to choose short-term MCS devices over more durable options to increase the allocation priority rather than medical needs [6]. The policy update also has the potential to cause earlier and more widespread use of short-term MCS.

Although many devices are available for similar indications, the choice of device ultimately depends on patient characteristics and provider and center familiarity as well as expertise with the device. A “shock team” as well as standard shock protocols can result in optimal utilization of MCS and improved CS outcomes [7, 8]. A shock team is comprised of invasive cardiologists, advanced heart failure cardiologists, cardiothoracic surgeons, and cardiovascular intensivists who convene when a patient with suspected cardiogenic shock is identified to discuss management, including further evaluation with right and/or left heart catheterization, medical support with inotropes and/or pressors, hemodynamic monitoring, and the potential need for percutaneous or surgical mechanical circulatory support [9]. Multidisciplinary shock teams result in the earlier and more appropriate use of mechanical circulatory support [10]. This review will cover the different types of temporary mechanical circulatory devices available and discuss their indications, contraindications, possible complications and management of complications, cannulations and connections, effects on hemodynamics, required monitoring and weaning from MCS.

## 2 LEFT VENTRICLE (LV) MECHANICAL SUPPORT

### 2.1 Intraaortic Balloon Pump

**Fig. (1):**
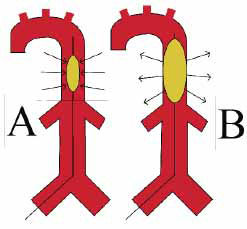
Intra-aortic balloon pump in systole (**A**) and diastole (**B**).

The intra-aortic balloon pump (IABP) was the first MCS device in contemporary use and remains one of the most widely used. The IABP consists of a 7-9 French (Fr) balloon catheter that is placed in the descending aorta *via* retrograde femoral artery access. The axillary artery is an alternative access site that can allow patients to ambulate. The balloon inflates during diastole to augment diastolic pressure and improve coronary perfusion (Fig. **1**). During systole, the balloon deflates to reduce left ventricular afterload. IABPs increase CO by approximately 0.5 L/min [11]. Once IABP placement is confirmed, its position must be confirmed daily with serial chest x-rays. Urine output and serum creatinine levels must be monitored as IABP displacement can cause occlusion of the renal arteries. Readjustment of the IABP can occur at the bedside.

Initial small randomized clinical trials (RCTs) of IABP use in CS demonstrated an average increase in CO from 4.8 ± 0.5 to 6.0 ± 0.5 L/min. However, the increase in CO was not significant when compared to the medication-only control group (3.6 ± 0.4 to 6.6 ± 0.6 L/min, *p =* NS) [12]. The larger subsequent IABP-SHOCK II trial revealed no benefit in 30-day (39.7% *vs* 41.3%, *p =* 0.69), 12-month (52% *vs* 51%, *p =* 0.91), or 6 years (66.3% *vs* 67.0%, *p =* 0.98) mortality in CS between the IABP and control groups [13, 14]. As a result, the IABP currently maintains a class III* recommendation for CS in European guidelines and an IIb** recommendation in United States (US) guidelines [15, 16].

At present, the American Heart Association (AHA) guidelines recommend temporary MCS as an adjunct to PCI in selected high-risk patients [17]. The first major RCT of IABP use in elective high-risk PCI did not demonstrate a significant reduction of in-hospital mortality (2.0% *vs* 0.7%, *p =* 0.34), 6-month mortality (4.6% *vs* 7.4%, *p =* 0.32), or major adverse cardiac and cardiovascular events (15.2% *vs* 16.0%, *p =* 0.85) compared to the control group [18].

In addition to the general MCS contraindications in Table **1**, IABP placement is contraindicated in patients with moderate to severe aortic regurgitation and severe aortic calcifications. The most common complications associated with IABP placement reported in IABP-SHOCK II include critical limb ischemia (4.3%), sepsis (15.7%), ischemic stroke (0.7%), and bleeding (20.6%) [19]. Vascular access complications (3.3%) and bleeding complications (19.2%) were reported with IABP in the high-risk PCI trial [18]. Management and prevention of these complications are listed in Table **3**.

* not useful/effective and may cause harm; evidence from a single randomized control trial (RCT) or large non-randomized trial

** usefulness/effectiveness uncertain or not well established, moderate quality evidence from 1 or more RCTs.

### 2.2 Impella

**Fig. (2):**
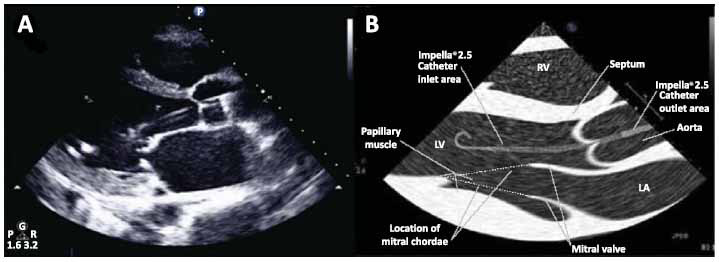
Parasternal long-axis view of Impella positioned in the left ventricle (adapted from Burzotta *et al.*, no changes were made to the original image) [20] (https://creativecommons.org/licenses/by-nc-nd/4.0/).

The Impella heart pumps are small, catheter-based left ventricle temporary mechanical support devices. The Impella system is available in different sizes, with varying insertion techniques and maximal flow capabilities. Details regarding the different Impella pumps can be found in Table **4**. The device is deployed in a retrograde fashion across the aortic valve, with an inflow tract positioned in the LV and an outflow tract in the ascending aorta (Fig. **2**). Using the principle of Archimedes’ screw, the device pumps blood from the LV to the aorta resulting in an unloading of LV volume. This leads to reduced wall stress and myocardial oxygen demand [20]. Additionally, the action of the Impella augments CO and causes an increase in diastolic pressure (and thus an increased mean arterial pressure) [21]. Indications for the use of the Impella vary based on the device. The Impella 2.5 and CP are approved for use in patients with CS within 48 hours of acute MI or open heart surgery, as well as in cardiomyopathy or LV failure that is not responsive to medical management. Furthermore, Impella 2.5 and CP also are approved for high-risk PCI. Impella 5.0 and 5.5 currently only have approval for use in patients with CS.

Several clinical trials have attempted to evaluate the safety and efficacy of the Impella in clinical practice. However, few of these studies have successfully been completed, and have often been limited by small sample size. The first such trial was the ISAR-SHOCK trial, which was a prospective, 2- center, randomized trial in which patients with CS secondary to acute myocardial infarction (AMI) were randomized to receive either IABP or Impella 2.5 device [22]. The study found that patients receiving Impella had a significantly greater increase in cardiac index 30 minutes postimplantation when compared to IABP (ΔCI = 0.49 ± 0.46 l/min/m2 *vs.* 0.11 ± 0.31 l/min/m^2^; *p =* 0.02). Furthermore, serum lactate levels were lower in Impella patients during the first 48 hours [area under the curve (AUC) = 123 ± 87 h°§mmol/l *vs.* 180 ± 147 h°§mmol/l] and time requiring mechanical ventilatory support was also lower [48 (6.7 to 147.8) hr *vs.* 98 (21.3 to 167.5) hr, *p =* 0.^15^]. The cardiac power index, a sign of LV workload, was significantly lower throughout the duration of device support in patients with Impella [4]. However, there was no difference in the overall use of inotropes. The study had a small sample size (*n =* 26), which limited its ability to evaluate mortality outcomes.

The IMPRESS in Severe Shock trial was a multicenter, open-label, randomized trial comparing Impella CP with IABP in patients with CS secondary to AMI [23]. This trial differed from ISAR-SHOCK in that the primary end-point was 30-day all-cause mortality, with a secondary end-point of 6-month mortality. In this trial, there was no significant difference in mortality at either 30 days (50% for IABP *vs.* 46% for Impella; hazard ratio [HR]: 0.96; 95% confidence interval [CI]: 0.42 to 2.18; *p =* 0.92) or 6 months (50% for both, HR: 1.04; 95% CI: 0.47 to 2.32; *p =* 0.92). As with ISAR-SHOCK, this study is also limited by a small sample size (*n =* 48).

Although there have been few randomized clinical trials, there have been several observational studies evaluating outcomes using Impella devices. Using the USpella registry, O’Neill *et al.* found that patients with CS secondary to AMI who received Impella had significant improvements in MAP (62.7±19.2 mmHg before Impella; 94.4 ± 23.1 mmHg after Impella; *p<*0.0001), cardiac index (1.9 ± 0.7 L/min before Impella; 2.7 ± 0.7 L/min after Impella; *p<*0.001), and CPO (0.48 ± 0.17 W before Impella; 1.0±0.48 W after Impella; *p<*0.0001) [24]. In addition, insertion of Impella prior to PCI allowed for the placement of more stents (1.94 ± 1.15 *vs.* 1.47 ± 0.85, *P =* 0.007) and treatment of more vessels (1.57 ± 0.67 *vs.* 1.30 ± 0.57, *p =* 0.01) when compared with placement after PCI. Placement of the Impella device prior to PCI in this study was found to be an independent predictor of improved survival until discharge (*p =* 0.01). Lauten *et al.* performed a retrospective analysis using the Impella-EUROSHOCK registry, which found that in patients receiving the Impella device, mean plasma lactate levels decreased from 5.8 ± 5.0 mmol/L to 2.5 ± 2.6 mmol/L (*p =* 0.023) within two days of placement [25].

Several retrospective analyses have attempted to compare Impella to other forms of MCS. A study utilizing data from 13 European hospitals and the EUROSHOCK registry found no difference in 30-day mortality when comparing patients receiving the Impella device with patients from the IABP-SHOCK II study (48.5% *vs.* 46.4%, *p =* 0.64) [26]. An additional retrospective analysis using the Impella-EUROSHOCK registry and the German Lifebridge registry evaluated outcomes in patients receiving Impella *vs.* ECMO [27]. Once more, there was no significant difference in 30-day mortality between the two groups (68% *vs.* 72%; OR: 0.84; 95% CI: 0.37–1.93; *p =* 0.69).

In addition to its use in CS, there are ongoing investigations into the preemptive use of Impella devices in patients undergoing high-risk cardiac surgery. LV dysfunction is a poor prognostic factor in patients undergoing cardiac surgery, with recent studies demonstrating operative mortality rates of approximately 7.5% [28]. Postcardiotomy shock, occurring in 2-6% of patients undergoing cardiac surgery [29], is most frequent in patients with reduced EF and is a detrimental development and catalyst towards multiorgan dysfunction and death. Historically, treatments such as inotropes, vasopressors, and afterload reduction using the IABP have been the mainstay of treatment for postcardiotomy shock. However, these therapies have heralded mixed outcomes and have their own inherent risks and adverse effect profiles [29]. Preemptive LV unloading using MCS in high-risk patients may allow for improved coronary blood flow, reduced myocardial oxygen consumption, and decreased wall stress, all of which may improve outcomes in patients undergoing cardiac surgery.

In a retrospective study evaluating outcomes in patients undergoing high-risk cardiac surgery with prophylactic impella support, survival to Impella removal and 30-day survival rate was 92.85% [30]. The median duration of Impella support was 4 days, with an average improvement in EF of 9%. Overall, data from this study show preemptive Impella implantation to be feasible and safe and pave the way for future research in this field. One such study is the IMPACT trial, which is an open-label, prospective, single-armed study evaluating the safety and effectiveness of peri-operative Impella 5.5 use in high-risk cardiac surgery patients. It is currently underway and is estimated to be completed in February 2024.

Despite the fact that there are very few data from large, randomized trials, the use of Impella devices in patients undergoing PCI in the United States has increased, with 31.9% of patients undergoing PCI requiring MCS using Impella in 2016 [27]. Absolute contraindications to the use of the Impella devices include the presence of a mechanical aortic valve and LV thrombus in addition to those listed in Table **1**, while aortic stenosis and regurgitation are relative contraindications [31]. Complications of Impella include those similar to other MCS devices (*i.e.* limb ischemia and bleeding at the insertion site) as well as complications specific to the device such as hemolysis secondary to shearing of red blood cells, device thrombosis, and renal failure [25]. As such, patients should be monitored for signs and symptoms of anemia, and hemoglobin (Hgb) and lactate dehydrogenase (LDH) levels should be monitored regularly. Renal function, including urine output, should be monitored as RBC lysis may lead to renal impairment mediated by pigment-induced tubular injury and is an indication for removal of the device [31]. Lastly, to prevent thrombosis formation in the device, systemic anticoagulation is recommended. A purge solution must flow in the retrograde to the direction of the blood being drawn into the catheter. This solution is comprised of unfractionated heparin and 5% dextrose in water (Table **5**).

### 2.3 iVAC

iVAC is a pulsatile, pneumatic catheter pump that consists of a bidirectional 17 Fr catheter with a two-way valve. iVAC is available as the first-generation iVAC3L and the second-generation iVAC2L. iVACL3L requires surgical cutdown of the axillary or subclavian artery for insertion. The iVAC2L can be inserted percutaneously *via* a transfemoral approach. The tip of the catheter is positioned in the LV and the two-way valve is located in the aorta. During systole, blood is aspirated from the LV into an extracorporeal pump containing two chambers. One chamber fills with blood and the other chamber is connected to an IABP console. During systole, the chamber connected to the IABP console fills with air, causing blood in the second chamber to propel back into the catheter. With this reversal in flow, the two-way valve changes position which results in expulsion of the blood into the aorta. The iVAC2L is capable of generating a CO of 2.0 L/min and the iVAC3L can produce CO of 2-3L/min [32-34].

Clinical experience with iVAC is limited and there are no randomized controlled trials of iVAC MCS. Additionally, iVAC has not been studied for use in cardiogenic shock. Most of the clinical experience with iVAC comes from use in high-risk PCI with the iVAC2L [35-38]. In 14 patients undergoing high-risk PCI with iVAC2L, 100% of patients achieved angiographic success [36]. The ongoing PULsecath entilator Support Evaluation (PULSE) trial (NCT032 00990) is a mechanistic exploratory study that aims to evaluate the hemodynamic effects of iVAC2L in patients with cardiogenic shock.

Given the limited experience with iVAC, the exact frequencies of complications are not known. Theoretical complications include access site complications, aortic valve injury, limb ischemia, major bleeding, infection, ischemic stroke, and hemolysis. The iVAC catheter crosses the aortic valve and is placed in the LV, therefore contraindications include the presence of an LV thrombus, severe aortic stenosis/calcification, mechanical aortic valve, and aortic insufficiency. Additional general MCS contraindications are listed in Table **1**. The iVAC cannot provide entilator support and is contraindicated in patients with combined circulatory and respiratory failure.

### 2.4 Tandem Heart

**Fig. (3):**
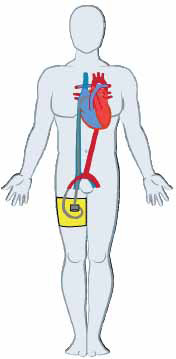
TandemHeart with inflow cannula in left atrium *via* the right femoral vein and transeptal puncture and outflow cannula in the right femoral artery.

Tandem Heart is a temporary MCS device consisting of an extracorporeal centrifugal pump, a 21 Fr inflow cannula, and a 15-17 Fr outflow cannula. The cannulas can be configured to provide either left or right ventricle MCS. To provide left ventricle MCS, the inflow cannula is percutaneously inserted in a femoral vein and placed in the left atrium *via* transseptal puncture while the outflow cannula is percutaneously inserted in a femoral artery (Fig. **3**). Oxygenated blood from the left atrium is suctioned and pumped into the femoral artery. TandemHeart is capable of providing up to 4 L/min of CO. In addition to the general MCS contraindications listed in Table **1**, TandemHeart is contraindicated in patients with inferior vena cava filter and severe aortic insufficiency.

In the initial RCTs of TandemHeart compared to IABP in cardiogenic shock, Thiele *et al.* reported TandemHeart had greater improvement in the cardiac index compared to IABP (1.0 *vs* 0.3 L/min/m^2^). TandemHeart had higher rates of complications such as limb ischemia (33% *vs* 0%, *p =* 0.009) and the need for blood transfusions (90% *vs* 40%, *p =* 0.002) [39]. Mortality was similar between the TandemHeart and IABP groups (43 *vs* 45%, *p =* 0.86) [36]. In a separate RCT comparing TandemHeart to IABP, Burkhoff *et al.* showed TandemHeart improved CO more than IABP (1.2 ± 0.8 *vs* 0.6 ± 0.6 L/min) [40]. However, TandemHeart and IABP had a similar rate of at least 1 major complication (94.7% *vs* 71.4%, *p =* 0.14) and mortality (47% *vs* 36%, *p =* NS) [40].

In the largest trial (*n =* 117) studying the efficacy and safety of TandemHeart in patients in CS, Kar *et al.* reported TandemHeart, on average, improved CI from 0.52 to 3.0 (*p<*0.001) [41]. The most common complications of TandemHeart were blood transfusions (58.90%), sepsis (29.90%), access site bleeding (29.05%), stroke (6.83%), groin hematoma (5.12%), and limb ischemia (3.14%) (Table **3**). Left atrial perforation occurred in one patient (0.85%). Thirty-day and 6-month mortality were 40.2% and 45.3%, respectively.

TandemHeart has also been studied for use in high-risk PCI. In a study comparing TandemHeart (*n =* 32) and Impella 2.5 (*n =* 36) for high-risk PCI, TandemHeart had a 99% success rate (*vs* 99% for Impella 2.5, *p =* 1.0) [42]. Rates of any in-hospital complication for TandemHeart was 12% (*vs* 28% for Impella 2.5, *p =* 0.14). The most common complications were access complications 6% (*vs* 8% for Impella 2.5, *p =* 0.48) and myocardial infarction 3% (*vs* 6% for Impella 2.5, *p =* 1.0). There was no reported death, stroke, hemolysis, or thrombocytopenia reported for either group during PCI. TandemHeart did not result in any renal failure (*vs* 6% for Impella 2.5, *p =* 0.18). 1 patient experienced left atrial perforation with TandemHeart insertion.

## 3 RIGHT VENTRICLE MECHANICAL SUPPORT

### 3.1 Impella RP

**Fig. (4):**
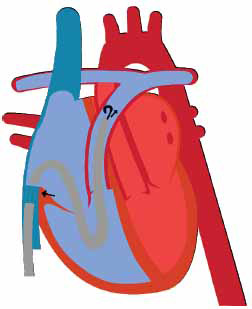
Impella RP with inflow tract in the IVC and outflow tract in the pulmonary artery.

The Impella RP is a percutaneously inserted axial pump for use in patients with acute right ventricular failure [43]. The 22 Fr motor is housed on an 11 Fr catheter. The catheter is inserted percutaneously *via* the femoral vein. The catheter then crosses the tricuspid and pulmonic valves with the outflow tract positioned in the pulmonary artery and the inflow tract positioned in the inferior vena cava (IVC) (Fig. **4**). Using the principle of Archimedes’ screw, blood is aspirated from the IVC and propelled into the pulmonary artery. The Impella RP is capable of generating flow from the IVC to the pulmonary artery at a maximum rate of 4.4 L/min [44]. The resultant reduction in RV pre-load leads to decreased right ventricular wall stress, decreased myocardial oxygen demand, and reduced systemic venous congestion, leading to improvement in CS and greater end-organ perfusion [43].

The RECOVER-RIGHT trial was a prospective, non-randomized multicenter study evaluating outcomes after insertion of the Impella RP device in patients with RV failure within 48 hours of LVAD placement and in those with RV failure within 48 hours of cardiac surgery or AMI [43]. Implantation of the Impella RP resulted in improvement in hemodynamic measures including increased CI (1.82 ± 0.04 to 3.3 ± 0.23 L/min/m^2^; *p<*0.001), and decreased central venous pressure (19.2 ± 0.7 to 12.6 ± 1 mmHg; *p<*0.0001). Survival to discharge occurred in 73.3% (22 of 30) of patients, and none of the patients discharged died within 180 days post-discharge. Limitations of this study include its small sample size (*n =* 30) and lack of a control arm [43]. The results of this trial led to a humanitarian device exemption allowing for use of the device [45]. The device has since received approval for providing RV support for up to 14 days in patients with RV failure following LVAD implantation, AMI, heart transplant, or open-heart surgery. Anderson *et al.* performed a prospective pooled analysis of data from the pre- and post- FDA approval studies for the Impella RP, which again demonstrated improvement in hemodynamics demonstrated by increased CI (1.9 ± 0.1 to 3.1 ± 0.1 L/min/m^2^; *p<*0.0001) and decreased central venous pressure (19 ± 0.8 to 13 ± 0.7 mmHg; *p<*0.0001) allowing for rapid weaning of inotropic/vasopressor support [17]. In this pooled analysis, 73.3% of patients (44 of 60) met the primary end-point of 30-day survival, with 62.4% overall survival after 180 days.

Contraindications to Impella RP include the presence of mechanical tricuspid or pulmonary valves, RV thrombus, mural thrombus of the right atrium or vena cava, presence of an IVC filter, or severe tricuspid or pulmonary valvular stenosis or regurgitation. The most common complications associated with Impella RV included bleeding and hemolysis [46].

### 3.2 TandemHeart RV

For TandemHeart to provide the right ventricle MCS, the 21F inflow cannula is placed in the right atrium and the 15-17F outflow cannula is placed in the PA. Most commonly, the outflow cannula is inserted percutaneously *via* the right femoral vein and the inflow cannula is inserted percutaneously *via* the left femoral vein. Alternatively, the outflow cannula can be inserted in the right internal jugular vein while the inflow cannula is inserted in either of the femoral veins. Deoxygenated blood from the right atrium is drawn and pumped to the pulmonary artery. This results in volume unloading of the RV, reduced RV wall stress, and reduced RV oxygen demand. TandemHeart RV is indicated for right ventricular failure. Moreover, its use has been reported in RV failure following myocardial infarction, severe pulmonary hypertension, acute mitral regurgitation, and post-LVAD implementation [47-50]. Contraindications to TandemHeart RV include general MCS contraindications listed in Table **1**, inferior vena cava filter, severe tricuspid or pulmonary valve stenosis, mechanical tricuspid or pulmonary valves, and right ventricular thrombi.

The TandemHeart RV is capable of generating 4L/min of CO. In TandemHeart Right Ventricular Support (THRIVE), the largest retrospective study of TandemHeart for right ventricular support, the average increase in CI was from 1.7 ± 0.7 to 2.2 ± 0.6 l/min/m^2^, *p =* 0.01 [51]. Overall in-hospital mortality was 57%. The most common complication was major bleeding (44%). Other complication risks of TandemHeart RV include vascular access complications, infection, ventricular arrythmias, cardiac perforation, and tamponade.

### 3.3 Protek Duo

**Fig. (5):**
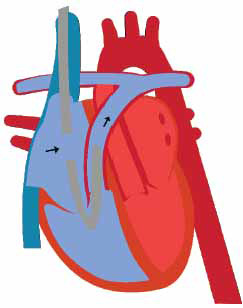
ProtekDuo right mechanical circulatory support device with inflow outlet in the right atrium and outflow outlet in the pulmonary artery.

The Protek Duo is a right ventricular support device with an extracorporeal centrifugal pump and dual lumen catheter. The dual lumen catheter is available as 29 or 31 Fr and is introduced *via* the right internal jugular vein. One benefit of internal jugular cannulation compared to femoral cannulation is early ambulation. One lumen contains inflow ports, which are positioned in the right atrium and the other lumen contains outflow ports, which are positioned in the PA (Fig. **5**). Dexogyneated blood is aspirated from the right atrium and pumped to the PA, which results in RV unloading, decreased RV wall stress, and decreased RV oxygen demand. Contraindications to the use of Protek Duo include mechanical tricuspid or pulmonary valves, severe pulmonary or tricuspid regurgitation/stenosis, right ventricular thrombi, and general MCS contraindications, as listed in Table **1**.

There are no randomized clinical trials of Protek Duo MCS. The majority of reported cases of Protek Duo implementation are in patients with left ventricular assist devices and right ventricular failure [52, 53]. In these case series, overall mortality was between 15-41%. The most commonly reported complications were bleeding and vascular access complications. Additional potential risks associated with the use of the Protek Duo include perforation of the myocardial wall, thrombus formation, myocardial infarction, pulmonary embolism, cardiac arrhythmias, vascular injury, major bleeding, infection, and renal impairment.

Given the internal jugular cannulation for the Protek Duo, one unique complication that has been reported is superior vena cava syndrome [54]. Signs of superior vena cava syndrome include facial plethora, distended and non-pulsatile neck veins, distended thoracic veins, dyspnea, and headache. There is an increased risk for SVC syndrome with Protek Duo when chronic indwelling central lines or ICD are in place and cause SVC stenosis. Management of SVC syndrome following percutaneous Protek Duo placement is revision with a central, surgically placed right ventricular assist device [54].

## 4 BIVENTRICULAR MECHANICAL CIRCULATORY SUPPORT

### 4.1 VA-ECMO

Veno-arterial extracorporeal membrane oxygenation (VA-ECMO) provides robust biventricular circulatory support. Unlike other forms of MCS, VA-ECMO is also capable of providing respiratory support and is the MCS of choice in patients with combined cardiopulmonary failure. VA-ECMO is comprised of inflow and outflow cannulations, an extracorporeal centrifugal pump, and an extracorporeal membrane oxygenator [55]. VA-ECMO inflow cannulas range in size from 19-25 Fr and VA-ECMO outflow cannulas range in size from 15-24 Fr. The cannulas can be inserted centrally or peripherally. Central cannulation requires surgical cut-down of the chest wall, with the inflow cannula directly inserted into the right atrium and the outflow cannula directly inserted into the aorta, subclavian artery, or pulmonary artery. Peripheral cannulation can be achieved percutaneously with the inflow cannula positioned in the right atrium or IVC *via* the femoral vein or the right internal jugular vein. The outflow cannula is positioned in the femoral or axillary artery.

**Fig. (6):**
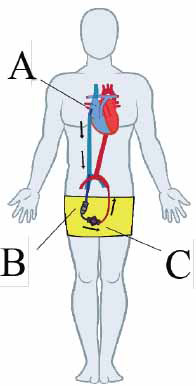
Peripheral venoarterial extracorporeal membrane oxygenation cannulations (**A**) with inflow cannula in the right atrium and outflow cannula in the left femoral artery. Mechanical pump (**B**) and membrane oxygenator (**C**).

With VA-ECMO, deoxygenated blood is aspirated from the RA or IVC and pumped through the centrifugal pump to the extracorporeal membrane oxygenator. Oxygenated blood is then returned to the femoral or axillary artery (Fig. **6**). If blood is returned to the femoral artery, it flows retrograde to the aorta to perfuse the head and upper extremities and flows antegrade to perfuse the lower extremities. If blood is returned to the axillary artery, it flows retrograde to the aorta to perfuse the upper and lower extremities.

VA-ECMO is capable of providing >4.5 L/min of cardiac output depending on the extracorporeal pump. Compared to other MCS devices, VA-ECMO has unique hemodynamic effects [56]. The inflow cannula in the right atrium unloads the right ventricle and decreases blood flow to the pulmonary circulation. Retrograde flow of blood from the outflow cannula into the aorta increases LV afterload. Elevated LV afterload results in decreased stroke volume as well as increased LV pre-load and pulmonary capillary wedge pressure.

In a meta-analysis of 29,289 patients with CS on VA-ECMO, a pooled short-term (30-day or mortality prior to discharge) was calculated at 61% (95% CI 59-63%) [57]. However the mortality varied widely based on the etiology of CS: post-cardiac transplant 35% (95% CI 29-42%), myocarditis 40% (95% CI 33-46%), pulmonary embolism 52% (95% CI 36-66%), decompensated heart failure 53% (95% CI 46-59%), post-cardiotomy 59% (95% CI56-63%), post-myocardial infarction 60% (95% CI 57-64%), in-hospital cardiac arrest 64% (95% CI 59-69%), and out-of-hospital cardiac arrest 75% (95% CI 69-83%) [57].

In 2018, the Organ Procurement and Transplant Network updated the adult heart allocation policy to reduce overcrowding in the highest priority tier [5]. Patients with modern LVADs have similar morbidity and mortality risk compared to patients in the lowest priority tier [58]. Therefore, the updated policy redistributed patients supported by LVADs to lower tiers, while patients requiring VA-ECMO remained in the highest priority tier [59]. This has led to an uptick in the number of patients receiving ECMO as a bridge to transplant [60]. In the months following the policy change (November 2018 to June 2019), the number of patients being supported by VA-ECMO at the time of listing increased from 1.2 to 3.2% [60]. As of March 2020, the number of patients being listed while being supported by VA-ECMO was 2.8% [61]. This change has led to a decrease in mortality for wait-listed patients as well as an increased number of patients receiving the transplant. Furthermore, data suggest that 30- and 180-day post-transplant mortality is improved in patients who utilized ECMO support as a bridge to transplant [62]. Additional studies on use of VAECMO to support patients with CS awaiting transplant are needed to help improve guideline recommendations for the use of MCS devices as a bridge to transplant.

Contraindications to VA-ECMO include general MCS contraindications listed in Table **1**, aortic dissection, and severe aortic insufficiency. VA-ECMO complications are common. In a meta-analysis of 20 studies comprising 1,866 patients on VA-ECMO for a cardiogenic shock, the most prevalent complications were significant bleeding (40.8%), infection (30.4%), lower extremity ischemia (16.9%), and stroke (5.9%) [63]. Additional risks of VA-ECMO include pulmonary hemorrhage, cardiac thrombosis, and coronary and cerebral hypoxia.

In a single-center study on 269 patients who received short-term MCS prior to implantation of an LVAD, patients on VA-ECMO had the lowest survival of all forms of temporary MCS [64]. Small trials have demonstrated benefits in the early transition from VA-ECMO to Impella 5.0 [65, 66]. Imepella 5.0 *via* cannulation of the axillary artery and decannulation of the VA-ECMO from the femoral artery is a reasonable option to promote early ambulation and has been associated with decreased complications associated with VA-ECMO. Another benefit of transitioning to Impella is for evaluation and optimization of RV function as VA-ECMO decompresses the right ventricle and can interfere with the assessment of RV function.

Two unique complications of VA-ECMO include LV distention and Harlequin syndrome. Retrograde flow of blood from the extracorporeal pump to the ascending aorta increases systemic circulation afterload. Elevated afterload results in LV volume overload and distention as well as increased LV wall stress and LV oxygen demand [67]. LV distention impairs LV recovery and in turn, can result in additional complications, such as pulmonary edema and LV stasis, leading to thrombosis [68]. LV distention is a common complication, occurring in as many as 70% of patients on VA-ECMO [69]. Patients on VA-ECMO should be monitored with daily echocardiography to evaluate for signs of LV distention. LV venting with IABP or Impella is a strategy that can be used to treat LV distention once identified or implemented prophylactically to prevent complications.

IABPs reduce afterload and have been studied in concurrent use with VA-ECMO. However, meta-analyses comparing outcomes of patients with CS on VA-ECMO with and without IABP have produced mixed results. Chen *et al.* in an analysis of 1,517 patients did not find IABP to reduce mortality in patients on VA-ECMO for CS (64.7% *vs* 62.5%; RR 0.875, 95% CI [0.745-1.261]; *p =* .10) [70]. Li *et al.* in a larger meta-analysis of 4,576 patients found IABP reduced in-hospital mortality (58.4% *vs* 63.1%; RR 0.9, 95% CI [0.85–0.95]; *p<*0.0001) without increased risk of complications [71].

Impella for LV venting has also been evaluated with VA-ECMO in a strategy referred to as “ECMELLA.” In the first large retrospective study evaluating Impella compared to propensity-matched controls for patients with cardiogenic shock on VA-ECMO, Impella was found to significantly reduce mortality (47% *vs* 80%, *p<*0.001) with increased rates of continuous venovenous hemofiltration (48% *vs* 19%, *p =* 0.02) and hemolysis (76% *vs* 33%, *p =* 0.004) [72]. In another retrospective study comparing survival rates of patients in CS on VA-ECMO and Impella to expected survival from Survival After Veno-arterial extracorporeal membrane oxygenation (SAVE) scores and Simplified Acute Physiology Score II (SAPS-II), Imeplla use was shown to have higher 30-day survival than the SAVE or SAPS-II predictions (35.8% *vs* 20% *vs* 6.9%) [73]. The most common complications of combined VA-ECMO and Impella use include the need for renal replacement therapy (59.4%), hemolysis (47.1%), sepsis (41.9%), vascular complication (34.3%), bleeding (24.8%), hypoxic brain injury (19.1%), and stroke (11.4%) [73]. When using Impella for LV venting, the goal is to unload the left ventricle and not improve CO. Therefore the Impella is typically run at the lowest power setting to accomplish LV unloading. Currently, there are no randomized control trials comparing VA-ECMO venting strategies, and more research in this area is needed.

**Fig. (7):**
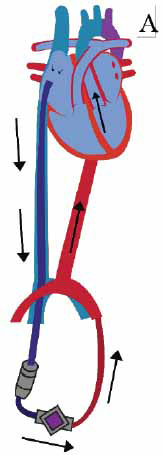
Depiction of “North-South Syndrome/Harlequin Syndrome” with VA-ECMO. The recovering heart pumps deoxygenated blood anterograde into the ascending and descending aorta. ECMO pumps oxygenated blood retrograde from the femoral artery into the descending aorta. At point (**A**), oxygenated and deoxygenated blood mix. The upper body and brain are perfused by deoxygenated blood and the lower body is perfused by oxygenated blood.

Harlequin syndrome, also known as a north-south syndrome, is another unique complication of peripheral VA-ECMO. When VA-ECMO is used for cardiopulmonary support, oxygenated blood flows retrograde from the femoral artery to perfuse the upper extremities and brain. However, when cardiac recovery occurs prior to recovery in pulmonary function, the left ventricle can overcome the retrograde flow of oxygenated blood from the VA-ECMO femoral artery cannula. When this occurs, deoxygenated blood from the non-functioning lungs is pumped to the upper extremities and brain (Fig. **7**). The most serious complication of the north-south syndrome is an anoxic brain injury. Monitoring for north-south syndrome requires close surveillance of oxygen saturation with pulse oximetry and blood gas sampling from the right upper extremity. The brachiocephalic artery, the first division of the ascending aorta, supplies the right subclavian artery and right common carotid artery and is the first vessel to receive deoxygenated blood from the recovering left ventricle. Once identified, there are several strategies to address north-south syndrome, including conversion to central VA-ECMO cannulation, veno-veno-arterial ECMO (VVA-ECMO), veno arterial venous ECMO (VAV ECMO), or venovenous ECMO (VV ECMO) [74]. In central VA-ECMO cannulation, the outflow tract is surgically placed proximally in the ascending aorta and does not have to overcome anterograde flow from the left ventricle. With VVA ECMO, an additional inflow cannula is placed in the right jugular vein, which reduces right and left ventricular preload as well as the volume of deoxygenated blood ejected from the left ventricle. Veno arterial venous ECMO requires an additional outflow cannula, which is placed in the internal jugular vein, allowing delivery of oxygenated blood to the brain. Lastly, if cardiac function recovers but pulmonary support is still required, transition to VV ECMO with the inflow and outflow cannulas placed in varying venous configurations can be considered to provide pulmonary support without concurrent cardiac support.

### 4.2 CardioHELP

The CardioHELP device is a small, portable ECMO system intended to be used for circulatory and/or pulmonary support for up to 6 hours during procedures requiring cardiopulmonary bypass, patient transport, or cardiac surgery. The device can provide up to 3.5 L/min of CO. Indications and contraindications are similar to those described previously in the ECMO section. The advantages of this device include its quick and simple initiation, ease of use, small device size and ease of portability, and the ability to manage patients without a perfusionist [75].

A retrospective study evaluating outcomes in patients (*n =* 15) who received the CardioHELP device between 2014 to 2017 during high-risk PCI found that all patients had successful PCI [75]. Three of the 15 patients suffered in-hospital mortality, and 3 suffered from additional major adverse cardiac or cerebrovascular events. Lastly, 7 patients required blood transfusion [75]. Adverse events with CardioHELP are similar to those described for the ECMO device previously.

### 4.3 CentriMag

The CentriMag device is a non-percutaneous external magnetically levitated centrifugal pump. It is indicated for temporary circulatory support of up to 30 days for uni- or bi-ventricular support. It has been used for patients with CS both pre- and post-cardiotomy with both its VAD and ECMO modes. The device has an inflow cannula attached to a magnetically levitated impeller through which blood flows and is returned through an outflow cannula positioned usually in either the main pulmonary artery or ascending aorta depending on which side of the heart is being supported. The device is able to generate a flow rate of up to 10 L/min. Cannulation of the inflow and outflow tracts is usually performed *via* either sternotomy or thoracotomy. The addition of a membrane oxygenator to the device allows for ECMO capabilities in patients with cardiorespiratory failure.

Borisenko *et al.* performed a systematic review and meta-analysis of 53 observational studies reporting data regarding the safety and effectiveness of CentriMag from 2003 to 2012 [76]. The meta-analysis found that the survival of patients in pre-cardiotomy CS was 82% (95% CI 70-92), while it was 63% (95% CI 46-78) in post-cardiac surgery CS [76]. In addition, in patients with RV failure post-LVAD placement, survival was 83% (95% CI 73-92) [65]. The most common adverse events in the studies included in the meta-analysis were bleeding [28% (95% CI 23-32)], renal complications [28% (95% CI 22-36)], and infection [24% (95% CI 19-30)]. Other less commonly reported adverse events included thrombosis, hemolysis, and neurologic complications [76].

An additional retrospective analysis analyzing outcomes in 63 patients from 2005 to 2017 who received the CentriMag device as a bridge to decision found that survival to explant of the device was 65.1% and survival to discharge was 58.7% [77]. Thirty-day and 90-day survival rates were 71% and 62%, respectively, while 1-year, 5-year, and 10-year survival rates were 55%, 46%, and 23% [77]. Similarly, the most commonly reported complications included bleeding (38%), renal failure requiring dialysis (46%), and bacterial (37%) or fungal infections (24%) [77]. The main contraindication to CentriMag is bleeding or the presence of any contraindication to the use of heparin for anticoagulation [78].

### 4.4 Bipella

**Fig. (8):**
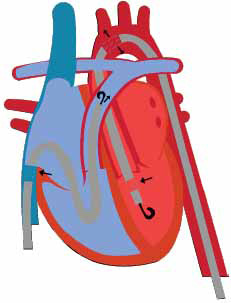
“BiPella” with Impella CP and Impella RP mechanical circulatory devices.

The use of an Impella device for LV support (either 2.5, 5.0, or CP) along with the Impella RP device for RV support is a novel method of biventricular support in refractory cardiogenic shock (Fig. **8**). Thus far, there have been no major clinical trials evaluating the effectiveness of simultaneous LV and RV support using two Impella devices. However, several case reports describing the use of Impella for biventricular support are reported. The first case was described in 2013 by Hunziker *et al.* in which a 54-year-old male developed biventricular failure secondary to AMI [79]. Implantation of a percutaneous LVAD improved pulmonary edema; however, right heart dysfunction, liver, and kidney failure persisted. After the placement of a right-sided Impella device, symptoms rapidly improved [79]. There have been several other reports describing the successful use of BiPella, as detailed in Table **5** [80-86]. The benefit of BiPella as opposed to other methods of Biventricular support, such as ECMO, is that the Impella device reduces, rather than increases LV afterload as well as requires a less significant degree of systemic anticoagulation compared to ECMO [80]. A retrospective analysis by Kuchibhotla *et al.* evaluated outcomes in 20 patients who received BiPella for the treatment of cardiogenic shock [76]. Among the 20 patients, 10 died during hospitalization. Of those who survived, BiPella led to improvements in cardiac index [1.8 (1.6-2.0) to 2.2 (1.7-2.7) L/min/m^2^], PCWP [23.1(20.2-26.1) to 19.3 (12.1-26.5)], pulmonary artery pulsatility index [0.9 (0.5-1.3) to 1.0 (0.7-1.4)] as well as several additional hemodynamic markers [76]. Complications of BiPella are similar to those of the Impella devices described above and include anemia requiring transfusion, limb ischemia, and intravascular hemolysis [79, 80].

## 5 WEANING MECHANICAL CIRCULATORY SUPPORT

To date, there are no clinical guidelines that provide a standardized approach to weaning off MCS. Therefore, weaning protocols may differ from institution to institution. Furthermore, weaning strategies may vary based on the device being used. Despite this, there are general principles that can guide the assessment of preparedness to wean. Evaluation of clinical, hemodynamic, metabolic, and imaging parameters should occur daily to identify patients who may be able to be weaned [87]. If a patient is deemed an appropriate candidate for a weaning trial, this can be performed to determine candidacy for explant of the device. These trials involve intentionally decreasing the amount of support provided by the device to determine whether the heart can meet the demand required of it for independent functioning. Some of the criteria to monitor that may help determine a patient's readiness to wean that have been proposed include MAP >65 mmHg and HR <100 bpm with less than moderate use of vasoactive agents, CI >2.2 L/min/m^2^, CVP ≤ 12 mmHG and PCWP ≤ 18 mmHg, lactate <2 mmol/L, evidence of adequate oxygenation and ventilation *via* arterial blood gas, and improvement in lab parameters assessing end-organ perfusions, such as transaminases, bilirubin, blood urea nitrogen, and creatinine [87]. In addition, evidence of improved ejection fraction to greater than 25% and improvement in valvular regurgitation should be considered [87]. If patients meet these criteria, a weaning trial can be performed. Trials should ideally include reducing the amount of support provided by the device (for example, reducing the ratio (frequency) of balloon opening in IABP, incrementally reducing or minimizing the support provided by Impella devices, or reducing ECMO or TandemHeart to flow of 2 L/min) and measuring the parameters previously described. If the patient continues to meet these parameters successfully, the device should be transitioned to the lowest level of support. It is important that patients be appropriately anticoagulated when reducing the flow rate of MCS, as the lower flow can increase the risk of thrombosis. If the patient continues to appear well clinically and hemodynamically, a team-based decision can be made regarding whether to proceed with explantation.

## CONCLUSION

Continued technological advancements have allowed MCS devices to take a larger role in the management of patients with heart failure. In addition, the utility of these devices has expanded, and they are now being implemented in high-risk PCI, treatment of acute circulatory failure following cardiac surgery, and bridging of end-stage heart failure patients. While MCS is being used more frequently, there is a paucity of large-scale randomized clinical trials evaluating the majority of these devices, and a large amount of the available evidence for their use comes from retrospective trials and clinical experience. In this review, we have attempted to highlight the available evidence supporting the use of the various MCS devices as well as provide an outline for considerations when using each device, including indications, contraindications, monitoring, and weaning protocols. Moving forward, further clinical trials may help pave the way for the development of additional guidelines regarding the use of MCS devices.

## Figures and Tables

**Table 1:** Cardiogenic shock etiologies and indications and contraindications for temporary mechanical circulatory support.

**Cardiogenic Shock Etiologies**
• Myocardial Infarction
• Decompensated Heart Failure
• Myocarditis
• Myocardial Contusion
• Drug-induced Cardiomyopathy
• Tachyarrhythmias
• Bradyarrhythmias
• Severe Aortic Insufficiency
• Severe Mitral Insufficiency
• Acute Valvular Rupture
• Valvular Abscess
• Critical Valvular Stenosis
• Rupture Ventricle Free Wall Aneurysm
• Severe Ventricular Septal Defect
• Atrial Myxoma
**Indications for Temporary Mechanical Circulatory Support**
• Cardiogenic Shock
• High-risk Percutaneous Intervention
• Bridge to LVAD or heart transplantation
• Acute rejection post-cardiac transplantation
• Unable to wean from cardiopulmonary bypass post-cardiotomy
• Cardiogenic Shock
**General Contraindications for Temporary Mechanical Support**
• Severe and irreversible neurological damage
• Severe peripheral vascular disease
• Severe uncontrolled infection
• Widespread malignancy
• Thrombocytopenia
• Inability to tolerate anti-coagulation

**Table 2:** Society of cardiovascular angiography and interventions cardiogenic shock staging [88].

**Stage**	**Description**	**Exam Findings**	**Labs**	**Hemodynamics**
A	Not currently in cardiogenic shock, but at risk for developing cardiogenic shock.	• Normal JVP • Lungs clear to auscultation • Strong pulses • Warm Extremities • Normal Mentation	• No laboratory abnormalities	Vital Signs: • Normotensive (SBP>100mmHg) Invasive Hemodynamics: • CI >2.5 • CVP <10 • PA sat >65%
B	Relative hypotension and tachycardia without evidence of hypoperfusion.	• Elevated JVP • Rales in lungs • Strong pulses • Warm Extremities • Normal mentation	• Normal lactate • No or minimal renal dysfunction • Elevated BNP	Vital Signs: • Hypotensive (SBP <90mmHg, MAP <60mmHg, or >30mmHg drop from baseline SBP) • Pulse >100 Invasive Hemodynamics: • CI ≥2.2 • PA sat >65%
C	Classic Cardiogenic Shock. Patients with hypoperfusion requiring intervention beyond fluid resuscitation (inotropes, temporary MCS, pressors).	Signs of Volume Overload: • Elevated JVP • Widespread rales Requiring ventilatory support (BiPAP, intubated) Signs of End-organ Malperfusion: • Cold, clammy extremities • Altered mental status • Decreased urinary output	• Lactate ≥2 • Creatinine doubles or >50% decline in eGFR • Increased LFTs • Elevated BNP	Vital Signs: • Hypotensive (SBP <90mmHg, MAP <60mmHg, or >30mmHg drop from baseline SBP) AND requiring inotropes/ pressors/ MCS to maintain BP targets Invasive Hemodynamics: • Cardiac index <2.2 • PCWP >15 • RAP/PCWP ≥0.8 • PAPI <1.85 • Cardiac power output ≤0.6
D	Similar to the patient in Stage C with a deteriorating clinic course.	Similar to Stage C	Similar to Stage C	Any of stage C requiring multiple pressors or the addition of MCS
E	Cardiac arrest with CPR and/or ECMO, requiring multiple interventions.	• Near pulselessness • Circulatory collapse • Mechanical ventilation • Defibrillation use	• CPR • pH ≤7.2 • Lactate ≥5	• No SBP without resuscitation • PEA or refractory VT/VF • Hypotension despite maximal support

**Table 3:** General complications of mechanical circulatory support.

**Complication**	**Prevention**	**Monitoring/Presentation**	**Management**
Hemorrhage	Micropuncture technique for device insertion	Monitor hemoglobin on complete blood count and for signs of clinical bleeding. May present with tachycardia or worsening hemodynamic parameters.	Transfuse blood products as needed.
Vascular Access Site Complications: Hematoma Fistula Pseudoaneurysm	Micropuncture technique as well as ultrasound and fluoroscopic guidance for device insertion	Check pulse at the access site and distally evaluate for swelling. Auscultate site for bruit. Monitor complete blood count.	Depends on severity, may simply require monitoring or surgical intervention.
Distal Limb Ischemia	Evaluate vascular access with ultrasound, CT, or angiogram prior to insertion of MCS Consider bypass circuits	Cold, blue, dusky extremities, Absent of peripheral pulses, Check distal pulses daily.	Revision of large bore access. Insertion of bypass circuit.
Infection	Sterile technique when inserting MCS	Redness around the catheter site. Increasing support or pressor requirements. Leukocytosis on complete blood count.	Obtain cultures and start broad-spectrum antibiotics. May require revision of invasive catheters.
Thrombocytopenia	Avoid MCS in patients with severe baseline thrombocytopenia	Can present as bleeding. Monitor platelets on daily complete blood count.	Transfuse platelets as needed.
Thrombosis	Systemic anticoagulation	Signs of stroke, critical limb ischemia.	Systemic anticoagulation.

**Table 4:** Characteristics of the different impella pumps for LV support.

**-**	**Impella 2.5**	**Impella CP**	**Impella 5.0**	**Impella 5.5**
Insertion	Percutaneously, through the axillary or femoral artery	Percutaneously, through the axillary or femoral artery	Surgically, through the axillary or femoral artery	Surgically, through the axillary or femoral artery
Catheter size	9 French	9 French	9 French	9 French
Motor	12 French	14 French	21 French	19 French
Max output	2.5 L/min	4.0 L/min	5.0 L/min	5.5 L/min

**Table 5:** Case Reports using BiPella for biventricular failure.

**-**	**Indication**	**Device**	**Days on BiPella**	**Outcomes**
Kapur *et al.* [80]	Stage D ischemic cardiomyopathy with moderate RV dysfunction	Impella 5.0 LP and Imeplla RP	5 days	-Increase in MAP from 65 mmHg to 78 mmHg - Improvement in mixed venous saturation from 42 to 68% -Decrease in serum creatinine 1.8 to 0.85 mg/dL
Aghili *et al.* [81]	Biventricular failure secondary to myocarditis	Impella CP and Impella RP	3 days	-MAP improved from 58 to 68 mmHg -Cardiac Index improved from 1.3 to 2.8 L/min/m^2^ -PCWP improved from 20 to 10 mmHg -RA pressure improved from 23 to 6 mmHg -BiPella was removed within 72 hours successfully
Kamioka *et al.* [82]	Biventricular failure secondary to non-ischemic cardiomyopathy	Impella 5.0 and Impella RP	6 days	-LV end-diastolic pressure improved from 33 to 20 mmHg -Central venous pressure improved from 26 to 15 mmHg -PCWP improved from 29 to 15 mmHg
Pappalardo *et al.* [83]	Biventricular failure and ischemic stroke status post thrombolytic therapy with TPA	Impella CP and Impella RP	7 days	-LV Ejection fraction improved from 10% pre-implantation to 49% on discharge (RV Ejection fraction 35% at discharge)
Chiu *et al.* [84]	Inferior NSTEMI complicated by cardiogenic shock	Impella CP and Impella RP	6 days	-Mixed venous O2 improved from 53% to 70% immediately -Inotropic and vasopressor support weaned completely by day 8 -LV ejection fraction improved from 40% on day three of BiPella to 61% by discharge
Dalal *et al.* [85]	Cardiogenic shock secondary to AMI	Impella 5.0 and Impella RP	3 days	-LV ejection fraction improved from 10% to 40% -Cardiac power output improved from 0.52 to 0.77 (normal >0.6) -Pulmonary artery pulsatility index improved from 0.7 to 1.0 (normal >0)
Zoltowska *et al.* [86]	Cardiogenic shock secondary to AMI	Impella CP and Impella RP	5 days	-Cardiac output increased from 1.22 to 3.6 L/min/m^2^ -Cardiac Power output improved from 0.33 to 1.49 -Pulmonary artery pulsatility index improved from 0.58 to 1.0

## LIST OF ABBREVIATIONS

MCS: Mechanical Circulatory Support
CS: Cardiogenic Shock
PCI: Percutaneous Coronary Intervention
LVAD: Left Ventricular Assist Devices
